# Bird Diversity and Bird-Strike Risk at Lincang Boshang Airport

**DOI:** 10.3390/ani15223250

**Published:** 2025-11-09

**Authors:** Jun Liu, Peng Liu, Jia Li, Jiansong Zhang, Yubao Duan

**Affiliations:** 1 Key Laboratory of Forest Resources Conservation and Utilization in the Southwest Mountains of China, Ministry of Education/Key Laboratory for Conserving Wildlife with Small Populations in Yunnan, College of Forestry, Southwest Forestry University, Kunming 650224, China; 2 Mengyang Administration Station, Xishuangbanna National Nature Reserve, Jinghong 666106, China; 3 Ecology Nature Conservation Institute, Chinese Academy of Forestry, Beijing 100091, China; 4 Southwest Survey and Planning lnstitute of National Forestry and Grassland Administration, Asian Elephant Research Center of National Forestry and Grassland Administration, Kunming 650000, China

**Keywords:** species diversity, phylogenetic diversity, functional diversity, airport birds

## Abstract

Accurate assessment of avian community structure and bird-strike risk within airport ecosystems is vital for balancing aviation safety with biodiversity conservation. From October 2019 to July 2020, we conducted systematic bird surveys at Lincang Boshang Airport (Yunnan, China) and its surrounding area. By integrating taxonomic, functional, and phylogenetic diversity analyses, we examined spatial–temporal patterns of bird diversity and characterized bird-strike risk. In total, 4859 individuals of 148 species were recorded, representing 51 families and 15 orders. The avifauna was dominated by broadly distributed Oriental–Palearctic species, reflecting the pronounced biogeographic transition of southwestern Yunnan. Functional diversity (FD) and phylogenetic diversity (PD) differed significantly among habitats: wetlands exhibited the highest FD and PD, indicating strong functional and lineage overdispersion driven by high environmental heterogeneity, whereas farmland showed the lowest FD and PD, consistent with stronger environmental filtering. Seasonal dynamics also shaped community structure, with the highest individual abundance in winter and the lowest species richness in spring. Standardized effect sizes (SES) revealed an overall tendency toward functional and phylogenetic clustering (SES < 0), most pronounced in forest and urban communities, while wetland assemblages consistently showed greater overdispersion across seasons. Risk evaluation indicated that low-risk species comprised 76.35% of the fauna, whereas high and very-high risk species accounted for only 3.38%, mainly large raptors (Accipitriformes) and pelicans/herons (Pelecaniformes). Integrating community patterns with risk distribution, we propose zone-specific management: remove standing water and tall grass in wetlands and farmland; optimize vegetation structure along forest–urban edges; and adopt acoustic/visual deterrents and dynamic management within core airport areas to reduce strike risk. Our findings provide a comprehensive baseline of airport bird diversity and bird-strike risk in southwestern China, offering evidence-based guidance for airport ecological safety management and regional biodiversity conservation.

## Simple Summary

Bird collisions with aircraft threaten flight safety, yet airports can also support many bird species. We surveyed birds at Lincang Boshang Airport in Yunnan, China, and nearby habitats across four seasons. We recorded 148 species and examined how different habitats—wetlands, forests, farmland, and urban areas—shape bird communities. Wetlands held the greatest variety of ecological roles (functional diversity) and the widest spread of evolutionary lineages (phylogenetic diversity), likely because they offer many types of food and shelter. Farmland showed the lowest values, suggesting that only species with similar needs can thrive there. Bird numbers peaked in winter, while spring had the fewest species. We also estimated which species could pose a higher risk to aircraft based on body size, flight height, where they occur, and how often they are seen. Most species were low risk; only a few large raptors and herons were high risk. We propose practical actions—reducing standing water and tall grass in high-risk areas, adjusting vegetation along habitat edges, and using non-lethal deterrents at the runway—to lower collision risk while protecting wildlife.

## 1. Introduction

Bird strikes refer to incidents in which birds or bats collide with aircraft, most commonly during takeoff and landing phases [1,2]. Such events not only pose potential threats to aircraft but also represent serious hazards to flight safety [3]. The first fatal bird-strike accident occurred in 1912 [4]. Since then, over more than a century of aviation development, the frequency of bird strikes has increased significantly in parallel with the expansion of global air transport [5,6,7], making it one of the major challenges in civil aviation safety worldwide. Studies have shown that bird strikes cause enormous annual economic losses to the global aviation industry [8,9,10] and pose severe threats to flight operations and passenger safety [11]. Consequently, the prevention and mitigation of bird-strike risks have become key concerns for international aviation authorities and researchers alike [1,12,13,14,15].

Airports, as essential components of air transportation infrastructure, provide open spaces and extensive grasslands that offer ideal foraging and roosting habitats for birds [1]. Some bird species have even adapted to intense human disturbances within airports, achieving higher foraging success in these low-competition environments [16]. In recent years, increasing attention has been paid to the factors influencing bird diversity and community structure in and around airports [17]. Variables such as crop type, vegetation composition, food availability, and landscape configuration have been shown to significantly alter bird community composition and distribution patterns [18,19,20]. This suggests that the types of habitats surrounding airports not only shape the distribution and behavior of bird populations but are also closely associated with bird-strike risk [21,22,23]. Accordingly, habitat characteristics within airport environments have gradually become integral to bird-strike risk assessment frameworks [24,25].

Research on how bird communities utilize different habitat types can help elucidate the relationship between habitat conditions and bird-strike risk mechanisms, providing theoretical support for airport landscape planning and ecological management [19,24,26]. Previous studies have demonstrated substantial variation in bird community structures among airports, and high-risk species often differ depending on geographic location and airport type [27]. Furthermore, avian biological traits are critical determinants of bird-strike risk. For instance, body mass influences the potential damage a species can inflict during collision—large-bodied waterbirds and raptors tend to cause more severe impacts [2]. Additionally, population size, spatial distribution, and seasonal migration patterns collectively affect the risk level of bird strikes [23,28].

Traditional biodiversity studies have largely focused on species richness, assuming that all species contribute equally to community structure, thereby neglecting differences in functional traits and evolutionary lineages [29]. Although this approach reveals taxonomic composition and species abundance, it provides limited insight into functional complementarity and community assembly mechanisms [30]. In contrast, functional diversity and phylogenetic diversity offer deeper ecological insights, especially for understanding community assembly, ecological niche complementarity, and evolutionary processes [31,32,33]. Functional trait diversity enhances ecosystem stability through resource-use efficiency and niche complementarity, helping to elucidate the processes driving biodiversity formation and maintenance [34]. When environmental filtering dominates, communities tend to exhibit functional and phylogenetic clustering, whereas stronger competitive exclusion leads to functional and phylogenetic overdispersion [35]. Studies have shown that habitat filtering typically governs community assembly in disturbed environments [36]. For example, avian communities in agricultural or semi-agricultural landscapes are mainly shaped by habitat filtering [37], while in resource-limited environments such as winter wetlands, interspecific competition and resource partitioning can significantly alter community structure [38]. Therefore, integrating analyses of functional and phylogenetic diversity provides a more comprehensive understanding of avian community characteristics and deeper insights into assembly mechanisms [39,40], offering new perspectives and methodological approaches for evaluating bird-strike risks in airport environments.

Based on this context, the present study analyzed bird community data collected from October 2019 to July 2020 in and around Lincang Boshang Airport, Yunnan Province, China. We compared avian community structures across four major habitat types—farmland, wetland, forest, and urban areas—by assessing taxonomic, functional, and phylogenetic diversity, and examined correlations among these indices. The study aimed to reveal patterns of avian community diversity and assembly processes under varying degrees of anthropogenic disturbance in airport environments, and to evaluate potential risk levels of different bird species. The findings not only enrich theoretical frameworks in community ecology and habitat utilization but also provide scientific support for avian management and bird-strike prevention strategies around airports.

## 2. Materials and Methods

### 2.1. Study Area

**Figure 1:**
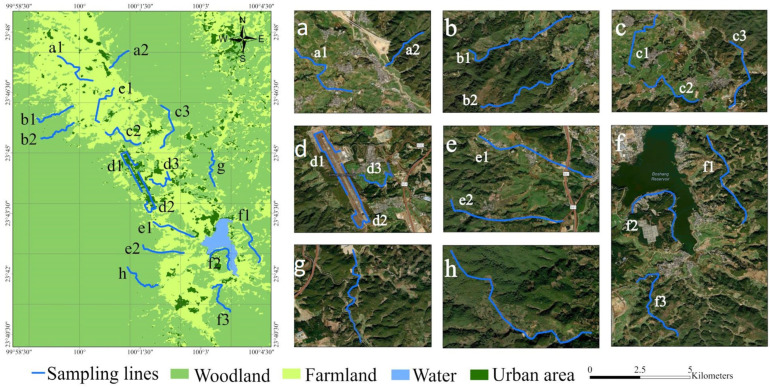
Schematic map showing the distribution of bird survey transects and habitat types in and around Lincang Boshang Airport. Panels (**a**–**h**) represent transects established across different habitat types (forest, farmland, wetland, and urban), with blue lines indicating specific survey routes. Each transect is labeled with a combination of letters and numbers. The main map illustrates the spatial distribution of transects and habitat types: light green represents forest areas, yellow-green denotes farmland, light blue indicates water bodies, and dark green corresponds to urban zones.

Lincang Boshang Airport is located on the western side of Boshang Town, Linxiang District, Lincang City, Yunnan Province, China (23°44′18″ N, 100°01′21″ E; elevation 1900 m) (Figure 1). The airport was completed and opened to traffic on 18 February 2001 [41]. It lies within the upper reaches of the Nanting River in Boshang Town. Due to intensive crustal uplift and fluvial erosion, the local terrain is highly undulating, sloping from north to south, with higher elevations in the west than in the east [42]. Boshang Town is situated in a narrow intermontane valley, with altitudes ranging from 1600 m to 2100 m.

The study area has a typical subtropical montane monsoon climate, characterized by abundant sunshine and plentiful rainfall. These favorable climate conditions foster diverse vegetation, and the forest coverage rate is approximately 47%. Among these, montane moist evergreen broad-leaved forest covers 27.9% of the total surveyed area and constitutes the dominant habitat behind the airport [43]. Other vegetation types include Yunnan pine forest, shrubland, and artificial tea plantations, together forming a complex ecological landscape pattern.

Geographically, Lincang Boshang Airport is located in the southern section of the Hengduan Mountain valley region, near the southern edge of the Nu Mountains and the headwaters of the Nanting River. The topography is rugged, and environmental heterogeneity is high. These unique geographic and ecological conditions provide diverse habitats for avian species and represent an important ecological background influencing the composition of bird communities in the airport area. The stable subtropical climate further promotes high avian diversity. Previous studies have surveyed bird communities in this region only during September–October, with limited spatial coverage [43]. Consequently, baseline data on avian communities around Lincang Airport remain incomplete. Comprehensive, year-round surveys are needed to enhance understanding of the local bird community and to provide scientific support for bird-strike prevention and ecological management at airports.

### 2.2. Bird Surveys

The survey area was determined in accordance with the Guidelines for the Ecological Environment Study of Birds at Civil Airports (AC-140-CA-2009-2) issued by the Civil Aviation Administration of China (CAAC). The study area was defined as the region within the outer boundary of the airport’s conical surface, corresponding to a radius of approximately 8 km around the airport. Landscape classification was based on Sentinel-2 remote-sensing imagery with a spatial resolution of 10 m (source: https://www.gscloud.cn (accessed on 14 February 2025)) and verified through field investigations. Within this range, four habitat types were delineated: urban, forest, farmland, and wetland. Supervised maximum likelihood classification was performed using ArcMap 10.8. Stratified sampling was then applied to establish transects within each habitat type. A total of 17 transects were set, each approximately 2.0 km in length and 100 m in width [44] (Figure 1).

Field surveys were conducted from October 2019 to July 2020, covering four seasons: autumn (October 2019), winter (December 2019), spring (March 2020), and summer (July 2020). Each seasonal survey lasted 7–10 days and included both airport interior and surrounding areas [45]. The line-transect method was employed [44]. Each survey team consisted of two members: one observer responsible for detecting and identifying birds, and the other responsible for recording the data and using the mobile application “GPS Toolbox (v2.3.5)” to determine the starting and ending coordinates of each transect. Observers walked along the centerline of the transect at a speed of 1.5–2.0 km/h, recording all birds detected within 50 m on either side using Asika 8 × 42 binoculars (Shuntu, Kunming, China). Recorded data included species identity, individual counts, group size, and flight altitude. All data were consistently collected by the same team members throughout the study, ensuring consistency and comparability across seasons. 

### 2.3. Data Analysis

#### 2.3.1. Species Accumulation Curves

To assess the sampling adequacy and representativeness of the bird community surveys, species accumulation curves (SACs) were used to evaluate species richness across survey areas [46]. SACs describe the relationship between cumulative sampling effort and the number of species recorded, allowing an assessment of whether the samples sufficiently represent the overall community composition. When the curve reaches an asymptote, it indicates that additional sampling yields few or no new species, suggesting that the survey has adequately captured the local bird diversity.

Furthermore, the expected species richness based on both incidence and abundance data was calculated to estimate the number of potentially unrecorded species [47]. This non-parametric rarefaction and extrapolation (R/E) approach, using estimators such as Chao1 and Chao2, enables standardized comparisons across samples with different effort levels, providing a more accurate reflection of true community diversity.

Sampling adequacy analysis was performed using the “vegan” package, version 2.7-2 [48] to compute species accumulation curves and the “iNEXT” package, version 3.0.2 [49] to plot rarefaction and extrapolation (R/E) curves, illustrating the relationship between observed and predicted species richness as well as sample coverage.

#### 2.3.2. Dominant Species Analysis

To identify the dominant taxa in bird communities across different habitats, this study employed the dominance assessment method proposed by Howes [50]. Dominance was evaluated by calculating the relative abundance of bird species, which is the percentage of individuals of a species in relation to the total number of individuals. The calculation formula is as follows:

$$Y=\frac{N_{i}}{N}\times 100\%$$

where *N_i_* is the number of individuals of species *i*, and *N* is the total number of individuals in the community. Based on the relative abundance *Y*, species with *Y* ≥ 10% are classified as dominant species.

#### 2.3.3. Calculation of Diversity Indices

(1) Taxonomic Diversity

Species diversity was quantified using species richness (SR), defined as the total number of bird species recorded within each transect. This index reflects the composition and fundamental structural characteristics of the community and serves as a foundational indicator in biodiversity and ecosystem studies.

(2) Functional Diversity

To evaluate differences in ecological functions among bird communities, functional diversity (FD) was calculated following the method proposed by Petchey and Gaston [51]. FD represents the total branch length of a functional dendrogram constructed within a functional trait space, reflecting the degree of functional dispersion among species. A higher FD value indicates greater functional differentiation among species and a more complex ecological functional structure.

A species-by-trait matrix was constructed, and Gower distances were computed to quantify pairwise dissimilarities among functional traits. Principal Coordinates Analysis (PCoA) was then used to reduce trait dimensionality. The convex hull volume of the reduced-dimensional trait space was estimated using the "FD" package [52] in R, version 1.0-12.3. A functional dendrogram was constructed using the Unweighted Pair Group Method with Arithmetic Mean (UPGMA), and FD values were computed using the “picante” package version 1.8.2 [53]. Four key functional traits were selected for this analysis: two continuous traits—body mass and hand-wing index (HWI), and two categorical traits—diet composition and vertical stratum use. All trait data were obtained from the Birds of the World database (https://birdsoftheworld.org (accessed on 14 February 2025)).

(3) Phylogenetic Diversity

Phylogenetic diversity (PD) was calculated using Faith’s PD [54], which represents the total branch length connecting all species within a community on a phylogenetic tree. This metric reflects the evolutionary breadth of a community’s species assemblage. In this study, 5000 Hackett phylogenetic trees were downloaded from birdtree.org [55]. A Maximum Clade Credibility (MCC) tree was generated using the TreeAnnotator module in BEAST software, version 1.10 [30,56]. The Faith’s PD values were then calculated using the “picante” package [53].

#### 2.3.4. Functional and Phylogenetic Community Structure

We used the mean pairwise functional distance (MFD) and mean pairwise phylogenetic distance (MPD) as indicators of community structure [31]. MFD and MPD represent the average pairwise functional or phylogenetic distance among all species within a community, calculated as follows:

$$MFD\;or\;MPD=\frac{\sum_{i\neq j}\delta_{\left(i,j\right)}}{n(n-1)}$$

where *n* is the species richness, and *δ_(i,j)_* represents the pairwise Euclidean distance between species *i* and *j*.

To assess whether the observed community structure significantly deviated from random expectations, we compared the observed values with the expected means derived from 999 randomly assembled communities. These random communities were generated by randomly shuffling species labels across the functional or phylogenetic distance matrices, while maintaining constant species richness within each habitat. The randomization procedure was performed using the functions ses.pd() and ses.mpd() in the R package picante, version 1.8.2 [49]. We then calculated the standardized effect size (SES) to quantify the deviation of observed community structure from random assembly:

$$SES=\frac{M_{obs}-M_{exp}}{{SD}_{exp}}$$

where *M_obs_* is the observed value, *M_exp_* is the mean of the 999 null model communities, and *SD_exp_* is their standard deviation. An *SES* value < 0 indicates functional or phylogenetic clustering, suggesting strong environmental filtering, whereas *SES* > 0 indicates functional or phylogenetic overdispersion, implying that competitive exclusion may be the dominant assembly mechanism [31].

#### 2.3.5. Calculation of Bird Risk Value

To assess the ecological status of bird species within the community and their potential threat to aviation safety, we calculated a bird risk value (R) based on six key ecological and behavioral indicators following the method of Ding et al. [57]. These indicators include abundance, flock size, spatial distribution, body mass, airport occurrence, and flight height.

The bird risk value was calculated using the following equation:

R=IV×F

where *R* represents the comprehensive risk value of a bird species, *IV* is the Importance Value, reflecting the relative ecological importance of the species within the community, and *F* is the Risk Coefficient, describing behavioral traits and the degree of spatial overlap between bird activities and aviation operations. Based on the magnitude of R, bird species were classified into four risk categories: *R* ≥ 25: extremely high-risk species; 15 ≤ *R* < 25: high-risk species; 5 ≤ *R* < 15: moderate-risk species; R < 5: low-risk species.

(1) Importance Value

The Importance Value (IV) represents the comprehensive ecological role of a species within the bird community and was calculated as follows:

$$IV=\frac{N+G+S+W}{4}$$

where

*N* = relative abundance index, calculated as (number of individuals of a species/maximum number of individuals) × 100;

*G* = relative flock size index, calculated as (average flock size of a species/maximum flock size) × 100, where flock size = number of individuals/encounter frequency;

*S* = spatial distribution index, calculated as (habitat area occupied by a species/total survey area) × 100;

*W* = body mass index, calculated as (average body mass of a species/maximum body mass) × 100.

(2) Risk Coefficient

The Risk Coefficient (F) quantifies the likelihood of spatial and altitude overlap between bird activity and aircraft operation, calculated as follows:

$$F=\frac{H+D}{2}$$

where *H* = flight height coefficient, classified into five levels according to the typical flight altitude range of the species: 0–5 m (0.1), 5–30 m (0.5), 30–50 m (1.0), 50–100 m (0.5), and >100 m (0.1).

This coefficient reflects the overlap risk between bird flight altitude and aircraft operation height. *D* = activity zone coefficient, quantifying the spatial overlap between bird activity areas and the airport zone: if a species was recorded within the airport, *D* = 0.5; if it occurred only in the peripheral airport cone zone, *D* = 0.1; if it occurred in both areas, the higher value (0.5) was applied. This composite index provides a quantitative framework to evaluate each species’ potential risk to aviation safety based on its ecological traits and spatial behavior.

#### 2.3.6. Data Analysis and Significance Testing

To compare differences in bird diversity indices among habitat types and seasons, non-parametric statistical tests were applied in this study. First, the Kruskal–Wallis test was used to examine overall differences in species diversity (SR, abundance), FD, PD, and community structure indices (MFD, MPD) across different habitats and seasons. When the overall difference was statistically significant (*p* < 0.05), pairwise Wilcoxon rank-sum tests were conducted for post hoc comparisons, with Bonferroni correction applied to control for multiple comparison errors. All statistical analyses were performed in the R 4.2.3 environment.

## 3. Results

### 3.1. Bird Species Composition

**Figure 2:**
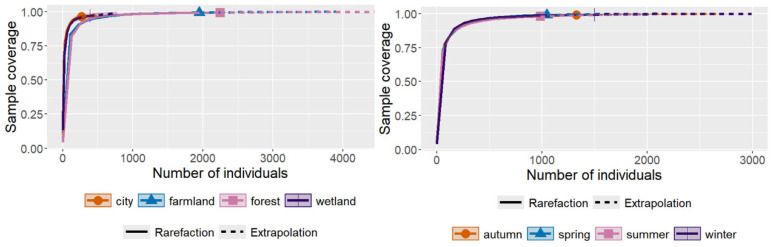
Sample coverage, rarefaction, and extrapolation curves of bird communities across different habitats and seasons. The (**left**) panel shows the rarefaction and extrapolation curves for four habitat types (urban, farmland, forest, and wetland). The (**right**) panel shows the curves for four seasons (spring, summer, autumn, and winter). Solid lines represent rarefied estimates, dashed lines represent extrapolated estimates, and symbols indicate the positions corresponding to actual sample sizes for each group.

The results of the species accumulation curves showed that the sampling coverage for all habitats and seasons exceeded 95% (Table 1; Figure 2), indicating that the sampling effort in this study was sufficient. A total of 148 bird species and 4859 individuals were recorded, belonging to 15 orders and 51 families (Table S1). The overall community was dominated by Passeriformes, with 33 families and 109 species, accounting for 73.65% of all recorded species and 64.71% of all individuals. This finding suggests that the bird community around Lincang Boshang Airport is mainly composed of small-sized, forest-dwelling, and omnivorous passerines, reflecting the characteristic assemblages of tropical montane bird communities.

**Figure 3:**
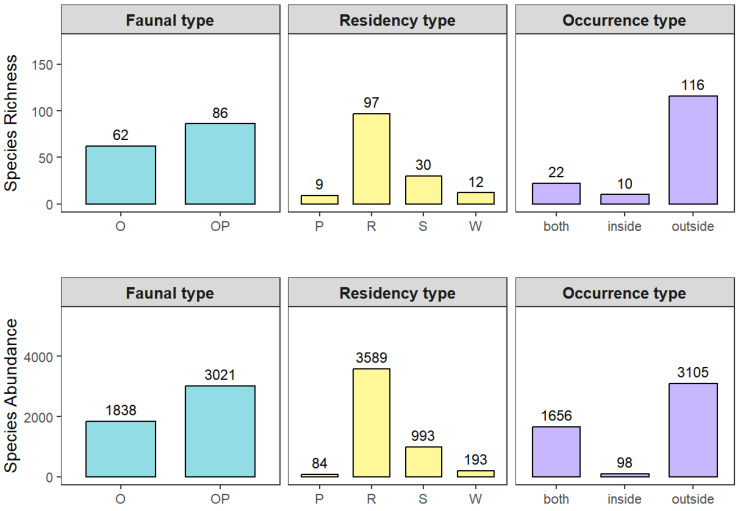
Bird species richness and individual abundance classified by zoogeographical type, residence type, and distribution type. The upper panel shows species richness, while the lower panel shows the number of individuals. Zoogeographical types include Oriental species (O) and Oriental–Palearctic species (OP). Residence types include passage migrants (P), residents (R), summer visitors (S), and winter visitors (W). Distribution types indicate the spatial occurrence of birds in relation to the airport area: both (inside and outside), inside (only within the airport), and outside (only outside the airport).

From a zoogeographical perspective (Figure 3), 62 species (41.89%) breed primarily within the Oriental realm, while 86 species (58.11%) have breeding ranges spanning both the Oriental and Palearctic realms. This indicates that the bird fauna in the study area is dominated by widely distributed species, exhibiting a distinct biogeographical transition between the two realms. Regarding residence types (Figure 3), resident birds were dominant, with 97 species (over 50% of all species) and 3589 individuals, accounting for 73.86% of the total individuals. This suggests a resident-dominated bird community structure in the study region.

### 3.2. Analysis of Dominant Bird Species in the Community

**Figure 4:**
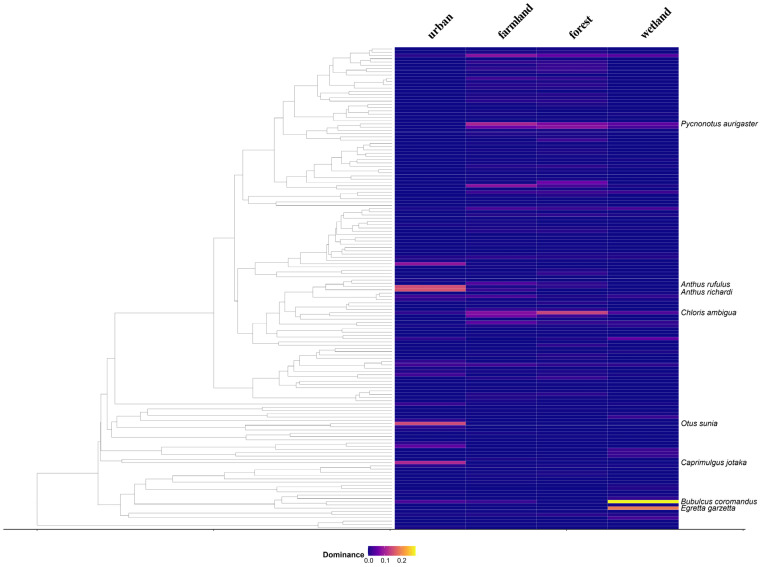
Phylogenetic tree and habitat dominance heatmap of bird species. The figure illustrates the phylogenetic relationships of 148 bird species and their relative dominance across four habitat types—urban, farmland, forest, and wetland. Color intensity indicates dominance values, with lighter colors representing higher dominance. Species with dominance values greater than 0.1 are labeled on the phylogenetic tree.

Based on the phylogenetic tree and habitat dominance heatmap (Figure 4), species exhibit distinct clustering patterns in both phylogenetic relationships and habitat dominance. Most bird species show relatively low dominance, indicating that they occur in small numbers across habitats. However, a few species display significantly higher dominance, suggesting their higher relative abundance in the habitats. Specifically, in farmland habitats, *Pycnonotus aurigaster* is the dominant species, showing the highest dominance, reflecting its adaptation to open agricultural landscapes. In wetland habitats, *Egretta garzetta* and *Bubulcus coromandus* dominate, representing typical wetland indicator species, which highlights the strong selective effect of wetland environments on wading birds. In forest habitats, *Chloris ambigua* is the main dominant species, indicating that the forest structure provides stable resources and shelter for its group activities. In urban habitats, *Anthus richardi*, *A. rufulus*, *Caprimulgus jotaka*, and *Otus sunia* are the main dominant species, frequently appearing in the areas along the airport runways.

### 3.3. Diversity Analysis Among Different Habitats

**Figure 5:**
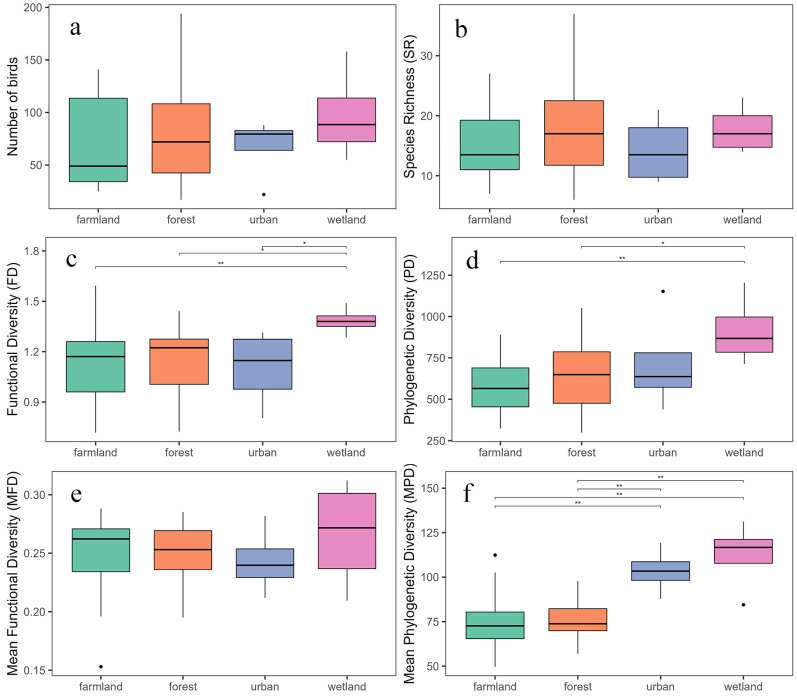
Comparison of bird community diversity among habitat types. Panels (**a**–**f**) show variations in species abundance (**a**), species richness (SR, (**b**)), functional diversity (FD, (**c**)), phylogenetic diversity (PD, (**d**)), mean pairwise functional distance (MFD, (**e**)), and mean pairwise phylogenetic distance (MPD, (**f**)) among four habitat types (urban, farmland, forest, and wetland). In boxplots, the black horizontal line represents the median, boxes represent interquartile ranges, whiskers indicate 1.5 × IQR, and solid dots denote outliers. Horizontal bars above boxes indicate significant differences between habitats (* *p* < 0.05, ** *p* < 0.01).

Among the four habitat types, the total number of bird species and individuals did not differ significantly (Figure 5a,b). However, both FD and PD showed significant variations across habitats (Figure 5c,d). In terms of FD, the wetland habitat exhibited the highest FD, significantly higher than that of forest (z = 2.501, *p* < 0.05) and urban areas (z = 2.089, *p* < 0.05), and extremely higher than farmland (z = 2.616, *p* < 0.01). Differences in FD among the remaining three habitats were not significant. This indicates that wetlands provide greater functional niche differentiation for birds, reflecting higher resource heterogeneity and habitat complexity. Regarding PD, wetlands also showed the highest PD, significantly higher than farmland (z = 2.616, *p* < 0.01) and forest (z = 2.013, *p* < 0.05), but not significantly different from urban areas. Differences among the other three habitats were also insignificant. This suggests that wetlands harbor not only greater phylogenetic richness but also bird assemblages from more diverse evolutionary lineages. MFD did not differ significantly among habitats, indicating that the overall distribution of birds in functional space remained relatively stable, without clear functional clustering or divergence patterns. In contrast, MPD differed significantly among habitats (Figure 5e,f). Wetlands had the highest MPD, significantly higher than farmland (z = 3.126, *p* < 0.01) and forest (z = 2.885, *p* < 0.01). Urban habitats ranked second, showing no significant difference from wetlands but significantly higher MPD than farmland (z = 3.000, *p* < 0.01) and forest (z = 2.760, *p* < 0.01). These results suggest that both wetlands and urban environments maintain relatively high levels of PD.

### 3.4. Seasonal Variation in Diversity

**Figure 6:**
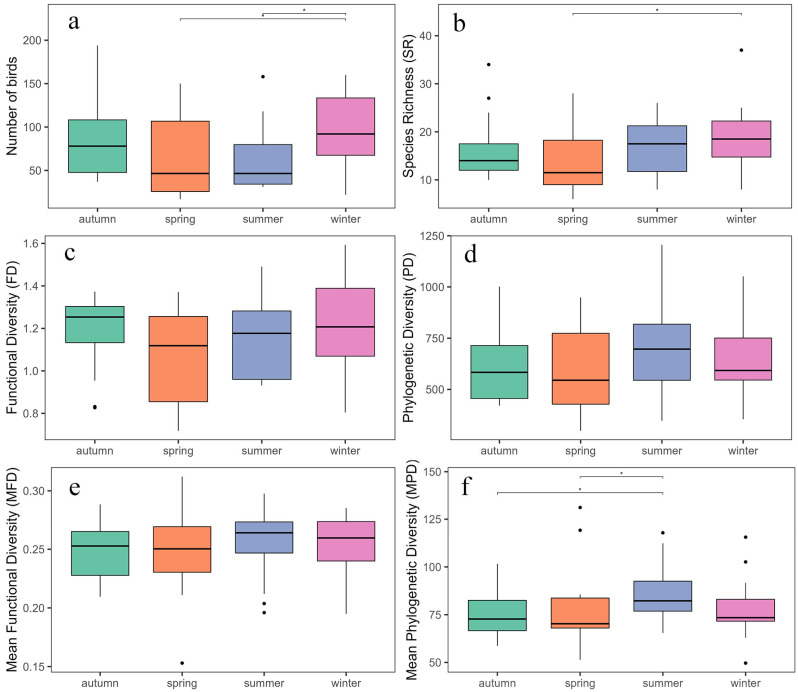
Seasonal variation in bird community diversity indices. Panels (**a**–**f**) show the variation in species abundance (**a**), species richness (SR, (**b**)), functional diversity (FD, (**c**)), phylogenetic diversity (PD, (**d**)), mean pairwise functional distance (MFD, (**e**)), and mean pairwise phylogenetic distance (MPD, (**f**)) across four seasons (autumn, spring, summer, and winter). In boxplots, black lines represent medians, boxes indicate interquartile ranges, whiskers extend to 1.5× IQR, and solid dots represent outliers. Horizontal bars above boxes indicate significant seasonal differences (* *p* < 0.05).

The number of bird species and individuals exhibited significant temporal variation across seasons (Figure 6a,b). The total number of individuals was highest in winter, significantly higher than in spring (z = 2.051, *p* < 0.05) and summer (z = 2.013, *p* < 0.05), while no significant differences were found between autumn and other seasons. Regarding species richness, spring had the lowest number of species, significantly lower than winter (z = 2.187, *p* < 0.05), but did not differ significantly from summer or autumn. FD and PD showed no significant seasonal differences (Figure 6c,d), indicating that the overall functional and phylogenetic composition of bird communities remained relatively stable throughout the year. Similarly, MFD did not vary significantly among seasons, suggesting a consistent functional structure in trait space. In contrast, MPD varied significantly among seasons (Figure 6e,f). The MPD value was highest in summer, significantly greater than in spring (z = 2.241, *p* < 0.05) and autumn (z = 2.032, *p* < 0.05), but not significantly different from winter. This result indicates that summer bird communities included more distantly related species, exhibiting a more divergent phylogenetic structure.

### 3.5. Community Assembly Analysis

**Figure 7:**
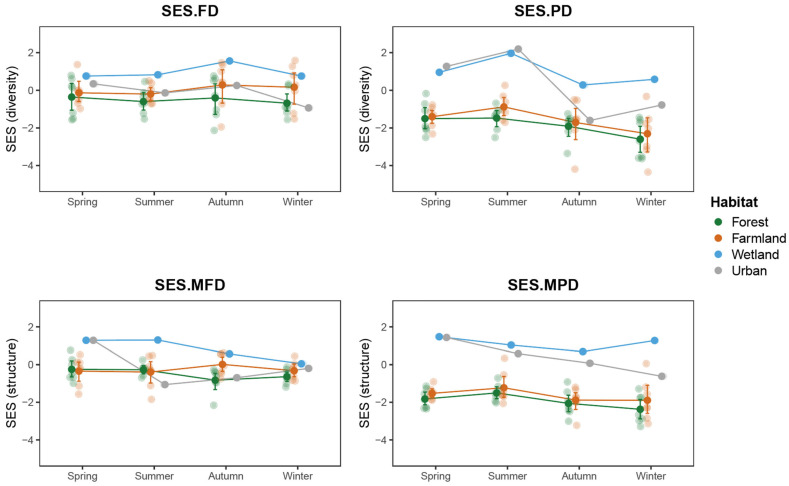
Seasonal variation in standardized effect size (SES) indices of functional and phylogenetic diversity across four habitat types. Each panel represents the standardized effect size (SES) for functional diversity (SES.FD), phylogenetic diversity (SES.PD), functional structure (SES.MFD), and phylogenetic structure (SES.MPD). Dots represent the mean (±SE) values of each habitat (forest, farmland, wetland, and urban) across four seasons (spring, summer, autumn, and winter). Transparent circles indicate observed values for individual transects.

Across different habitat types, both functional diversity (SES.FD) and phylogenetic diversity (SES.PD) exhibited similar seasonal variation patterns (Figure 7). The wetland community consistently maintained the highest SES values throughout the year, indicating a pronounced functional and phylogenetic overdispersion within this habitat. In contrast, the forest community showed significantly lower SES values in winter, suggesting a more clustered community structure and greater functional trait convergence, reflecting the dominant role of environmental filtering during this period. The SES values of farmland and urban habitats were intermediate and showed slight seasonal fluctuations, implying that these human-disturbed habitats may exhibit a degree of structural instability in both functional and phylogenetic composition. For the community structure indices (SES.MFD and SES.MPD), most values were negative, indicating an overall trend of functional and phylogenetic clustering, where species tend to be more similar in traits and evolutionary lineage. The forest community showed the strongest clustering, suggesting that its species composition is highly constrained by habitat conditions. In contrast, wetland communities displayed a relatively dispersed pattern across seasons, reflecting greater differentiation among species in terms of both functional traits and phylogenetic relationships.

### 3.6. Bird-Strike Risk Index Analysis

**Figure 8:**
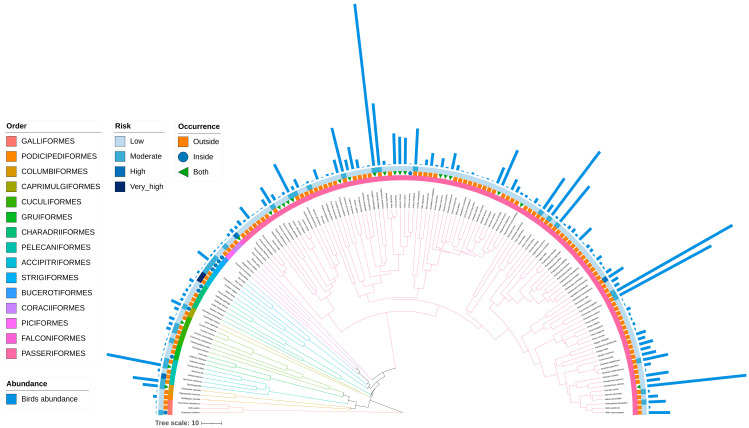
Phylogenetic distribution, abundance, spatial occurrence, and bird-strike risk of avian species recorded within and around Lincang Boshang Airport. Branch colors and the inner ring represent taxonomic orders. The second ring shows spatial distribution types: outside only (orange), inside only (blue), and both inside and outside (green). The third ring indicates bird-strike risk levels, from light to dark: low, moderate, high, and extremely high. The outermost bars represent relative abundance, showing the number of individuals of each species in the study area.

In total, 148 bird species recorded within and around Lincang Boshang Airport were evaluated for their bird-strike risk (Figure 8; Table S1). According to the classification criteria for bird-strike risk levels, the results showed the following: extremely high-risk species: 2 species (1.35%); high-risk species: 3 species (2.03%); moderate-risk species: 30 species (20.27%); low-risk species: 113 species (76.35%). Overall, the bird community was dominated by low-risk species, with only a few raptors and nocturnal species exhibiting higher potential risk to aviation safety. At the extremely high-risk level, *Pernis ptilorhynchus* and *Buteo japonicus*, both belonging to Accipitriformes, were mainly active along forest edges and farmland near the airport. Although their numbers were relatively low, their large body size, medium flight altitude, and wide activity range suggest that their presence in airport airspace could pose severe strike hazards. The high-risk level included three species from Falconiformes, Pelecaniformes, and Passeriformes. Among them, *Nycticorax nycticorax* and *Hypsipetes leucocephalus* occurred only in surrounding wetlands or forests, whereas *Falco tinnunculus* was distributed both inside and outside the airport area. Among the 30 moderate-risk species, Passeriformes dominated, accounting for 16 species (53.33%) and 2337 individuals (85.76%) within this risk level. Notably, *Lanius schach* and *C. ambigua* occurred both inside and outside the airport, demonstrating strong environmental adaptability. Other Passeriformes species mainly inhabited forest and farmland edges surrounding the airport. All recorded species from Strigiformes and Caprimulgiformes were classified as moderate-risk and were found exclusively within the airport. Their nocturnal activity patterns further increase potential flight safety concerns. The low-risk group contained the largest number of species (113), with Passeriformes again comprising the majority (73 species, 64.6%) and 1949 individuals (94.43%). These species were generally small, resident, and low-flying birds that rarely entered the core airspace of the airport, thus posing minimal threat to aviation safety.

## 4. Discussion

### 4.1. Composition Characteristics of Bird Communities and Regional Ecological Context

The avifaunal composition was dominated by species widely distributed across the Oriental and Palearctic regions, indicating a distinct tropical–temperate transitional nature of the bird community. This pattern is closely related to the geographical position and diverse topography of southwestern Yunnan [58,59]. Lincang lies within the transitional zone between the Hengduan Mountains and the northern Indochina Peninsula, characterized by a humid climate and clear altitudinal zonation, which provides suitable habitats for multiple ecological types of birds [43]. Consistent with findings from other regions of Yunnan [60,61], the bird community exhibits both tropical and temperate features, including typical forest-dwelling species as well as numerous open-land and urban-adapted species. This mixed faunal composition reflects the transitional biogeographic and ecological characteristics of the Lincang region and provides an important case for understanding the shift of tropical bird communities toward temperate assemblages.

### 4.2. Effects of Different Habitats on Avian Diversity

Wetlands exhibited the highest FD and PD values, suggesting that their communities possess greater functional and phylogenetic divergence. This may be attributed to the high heterogeneity and resource richness of wetland environments [30], where complex microhabitats provide a wide range of ecological niches for birds with different functional traits, thereby promoting trait diversification and phylogenetic dispersion. In contrast, farmland habitats showed markedly lower FD and PD values, reflecting stronger environmental filtering effects [31]. In such homogeneous and resource-limited habitats, species with similar ecological adaptations are more likely to coexist, leading to higher community clustering [62]. Overall, habitat heterogeneity is a key ecological factor shaping the spatial patterns of bird diversity. The complexity and continuity of wetland ecosystems help maintain higher levels of functional and phylogenetic diversity, whereas the simplified landscapes of farmlands and urban areas constrain ecological differentiation within bird communities.

### 4.3. Seasonal Variation and Community Structure Dynamics

Seasonal changes exerted significant effects on both the composition and functional structure of bird communities. The number of individuals was highest in winter, while species richness was lowest in spring, indicating that the arrival of winter migrants substantially expanded community size. The analysis of standardized effect size (SES) for functional and phylogenetic structure revealed that wetland communities maintained high SES values throughout the year, suggesting weaker environmental filtering and stronger niche differentiation. This pattern is likely associated with avian migratory strategies, as the influx of migratory species alters both the functional trait composition and phylogenetic structure of communities, leading to greater functional and phylogenetic dispersion [63]. Most SES.MFD and SES.MPD values were negative, indicating a general trend toward functional and phylogenetic clustering [64]. Such clustering likely arises from the combined effects of environmental filtering, resource distribution, and spatial constraints. In farmland and urban habitats, human disturbance and habitat fragmentation reduce ecological niche availability, thereby increasing the likelihood of coexistence among functionally similar species [65]. In contrast, wetland communities displayed greater divergence across seasons, suggesting that wetland environments support a higher degree of niche diversity and functional redundancy, accommodating a wider range of ecological strategies among bird species.

### 4.4. Patterns of Dominant Species and Ecological Adaptation

Through the analysis of habitat types and dominant species, we can see that the bird community structure in each habitat is largely determined by the habitat environment and species adaptation. For example, in farmland habitats, the *Pycnonotus aurigaster* shows adaptation to open landscapes, while in wetlands, species like *Egretta garzetta* and *Bubulcus coromandus* represent the selective pressure of wetland environments on wading birds. In urban habitats, species like *Anthus richardi* and *A. rufulus* are frequently found in the grassland areas along airport runways, likely because these areas provide suitable foraging resources; meanwhile, *Caprimulgus indicus* and *Otus sunia* are mainly recorded during the migratory season, possibly due to the light-attraction effect of the airport, especially at night, when airport lights may interfere with or attract these nocturnal species. Different habitats provide abundant food resources and shelter for species, resulting in dominant species displaying different ecological roles in various habitats, showing how species adapt and differentiate according to habitat characteristics. These distribution patterns not only demonstrate bird responses to human activities but also reveal the ecological filtering effects caused by urbanization and agricultural expansion. These ecological filtering effects are manifested as significant species ecological differentiation within bird communities in different habitats, reflecting the selective pressures and adaptive strategies imposed by habitats. This differentiation pattern helps us better understand how birds respond to habitat changes through functional roles and adaptive strategies.

### 4.5. Bird-Strike Risk Index and Management Implications

Based on the bird-strike risk index evaluation, the overall avian risk level around Lincang Boshang Airport was relatively low: low-risk species accounted for 76.35%, medium-risk species for approximately 20%, and high- to very high-risk species for only 3.38% of all recorded species. This suggests that although bird activity around the airport is frequent, relatively few species pose significant threats to aviation safety. High-risk species were mainly from Accipitriformes and Pelecaniformes, such as *Butastur indicus* and *N. nycticorax*. These large-bodied birds often fly at moderate altitudes and are active in open areas near the airport, posing potential safety hazards. Medium-risk species were dominated by Passeriformes (e.g., *L. schach*, *C. ambigua*), which are abundant and frequently recorded within the airport boundaries, thus warranting focused monitoring and management. Based on community structure and risk index patterns, a zonal and differentiated management strategy is recommended:

(1). Wetland and farmland zones—regularly remove standing water and tall grasses to reduce the attraction of wading birds.
(2). Forest and urban edge zones—optimize vegetation structure to decrease nesting opportunities for edge-dependent species.
(3). Airport core areas—implement dynamic deterrence measures, such as sound-light repellents, drone patrols, and time-segmented bird management to minimize aggregation risks.

Furthermore, long-term bird population monitoring should be integrated to understand how bird communities dynamically adapt under urbanization and aviation disturbances. Such data-driven approaches will provide scientific and technical support for balancing ecological integrity with aviation safety [14,25,28].

### 4.6. Limitations and Future Prospects

This study, based on annual transect monitoring and phylogenetic analyses, systematically revealed the diversity patterns and risk distribution of bird communities around Lincang Boshang Airport. However, due to temporal and observational constraints, interannual variations and nocturnal avian activities were not fully captured. Future research should incorporate automated acoustic monitoring, radar ornithology, and remote sensing techniques to conduct multi-temporal and multi-spatial analyses of community dynamics. Additionally, integrating climatic variability, land-use change, and aviation operation data will enable the construction of predictive bird-strike risk models, providing forward-looking scientific foundations for airport ecological management and aviation safety planning.

## Figures and Tables

**Table 1:** Species richness, abundance, and sample coverage of birds across different habitats and seasons.

	Species	Abundance	Coverage
urban	32	269	0.9629
farmland	94	1951	0.9928
forest	103	2249	0.9929
wetland	39	390	0.9693
autumn	86	1329	0.9895
spring	81	1048	0.9895
summer	88	985	0.9797
winter	84	1497	0.992

## Data Availability

The data that support the findings of this study are available in the Supplementary Materials of this article. Please contact the corresponding author directly as needed.

## Supplementary Materials

The following supporting information can be downloaded at: https://www.mdpi.com/article/10.3390/ani15223250/s1. Table S1: Origianl data.
